# How lizards fly: A novel type of wing in animals

**DOI:** 10.1371/journal.pone.0189573

**Published:** 2017-12-13

**Authors:** J. Maximilian Dehling

**Affiliations:** Zoology Group, Department of Biology, IfIN, University of Koblenz-Landau, Koblenz, Germany; Brown University, UNITED STATES

## Abstract

Flying lizards of the genus *Draco* are renowned for their gliding ability, using an aerofoil formed by winglike patagial membranes and supported by elongated thoracic ribs. It remains unknown, however, how these lizards manoeuvre during flight. Here, I present the results of a study on the aerial behaviour of Dussumier's Flying Lizard (*Draco dussumieri*) and show that *Draco* attaches the forelimbs to the leading edge of the patagium while airborne, forming a hitherto unknown type of composite wing. The attachment of the forelimbs to the patagium suggests that that aerofoil is controlled through movements of the forelimbs. One major advantage for the lizards is that the forelimbs retain their complete range of movement and functionality for climbing and running when not used as a part of the wing. These findings not only shed a new light on the flight of *Draco* but also have implications for the interpretation of gliding performance in fossil species.

## Introduction

A number of vertebrates and invertebrates are known to perform gliding flights [1–3]. Flying Lizards of the agamid genus *Draco* are the most specialized and best-studied gliding reptiles [2,4–6]. Their patagium is supported by five to seven greatly elongated thoracic ribs that are spread by specialized iliocostalis and intercostal muscles [1,3,4,6–8]. It is commonly assumed that flying lizards use the unfurled patagium to glide and hold the forelimbs free in front of the body while airborne. This assumption was promulgated about 300 years ago, when the first preserved specimens were brought to Europe and reports of flying lizards were accompanied by drawings showing artistic interpretations of them holding their forelimbs in front of the body while “gliding” [9–12]. The patagium-associated musculature has been suspected to be used to control the direction of the glide path [1,3,6–8], but it has remained unclear how the lizards are able to manoeuvre in the air [2,6].

Anatomical properties of the patagium, as well as behavioural observations, challenge the assumption that the associated muscles alone can perform the sophisticated movements required for manoeuvring: Only two muscles insert the first elongated ribs [7] and therefore can only produce movements in a limited number of directions, mainly those spreading (forward) and furling (backward) the patagium. The patagium-spreading muscles stem from musculature originally used for breathing [7,13]. In the ancestral state, the intercostal muscles of both sides contract simultaneously in order to expand and contract the thorax [14]. The patagium is spread not only to form an aerofoil but also for display in intraspecific communication, and photographs and observations of display of different species of *Draco* indicate that both sides of the patagium are moved simultaneously [6,15–18]. If *Draco* was able to perform the sophisticated unilateral movements required for glide-path control with their patagium-associated muscles alone, such movements would also probably be exhibited during display, which appears not to be the case. Therefore, it appears unlikely that the major component for flight control are the specialized trunk muscles. Here, I report on the results of a study I conducted on the aerial behaviour of Dussumier's Flying Lizard (*Draco dussumieri*) in order to investigate whether the patagium might be controlled in a different way. Observations and documentation of gliding flight in the animal’s habitat are supplemented by examinations of morphological characteristics of preserved specimens of *D*. *dussumieri* and 17 other species of *Draco*. My findings suggest that the patagium is actually controlled by the forelimbs and thus point to a hitherto unknown type of composite wing in animals.

## Material and methods

### Behavioural observations

I observed gliding flights of *Draco dussumieri* in areca nut (*Areca catechu*) and coconut (*Cocos nucifera*) plantations near the towns of Agumbe (13.509536°N, 75.097385°E, WGS 84; 650 m a.s.l.) and Hebri (13.458442°N, 74.990963°E; 80 m a.s.l.), Karnataka, southwestern India, during the late morning and early afternoon (9–15 h) on fourteen non-consecutive days in March 2015 and in March and April 2017. The observations were made using a non-experimental approach of the natural behaviour in the habitat, where the lizards performed gliding flights from one tree to another. No animal was captured, handled, or manipulated in any other way during the study. A total of approximately 500 gliding flights performed by at least twenty different males and six different females were observed, often with the aid of Minox 10x50 binoculars. The minimal total number of individuals results from the addition of the respective maximum numbers of individuals that were observed simultaneously at three different, geographically separated observation localities. Sequential short-exposure photographs of a total of about 150 glides were taken with Nikon full-frame digital single-lens reflex cameras D600 at a rate of 5.5 frames per second (March 2015) or D750 at 6.5 frames per second (March/April 2017), each equipped with a Nikon AF-S 200–400 mm telephoto zoom lens (manually focused). Video sequences of the composite wing formation and the landing were recorded at 60 frames per second with the same camera and lens equipment. Movements and actions were investigated by frame-by-frame analyses. Estimates of speed at a given phase of the gliding flight were made from four documentations by measuring the distance between two spots at which a lizard was photographed during a photo or video sequence with a measuring tape and dividing it by the time that had passed between the consecutive frames.

### Morphological examination

In order to corroborate observations of possible morphological adaptations for gliding flight in *Draco*, I examined voucher specimens of 18 species of *Draco* and 21 species of 12 representative genera of other arboreal Asian agamid lizards deposited in the herpetological collection of the Zoologisches Forschungsmuseum Alexander Koenig (ZFMK), Bonn, Germany (S1 Table). I took measurements (to the nearest 0.1 mm) with a digital calliper of snout-vent length (SVL, from tip of snout to vent), arm length (AL, from the forelimb insertion to the distal end of the antebrachium, measured with the arm extended perpendicularly to the median body plane), and length of the leading edge of the patagium (LL, from the insertion of the first elongated rib to the point at which the leading edge starts to bend posteriorly, i.e. the lateral margin of the leading edge; given as a percentage of the corresponding arm length, rounded to the nearest 1%). I checked the ability to deviate the wrist ulnarly (adduction) and radially (abduction) in all specimens, as well as the relative length of the fingers. The results of the examination are given in S1 Table.

## Results

**Fig 1:**
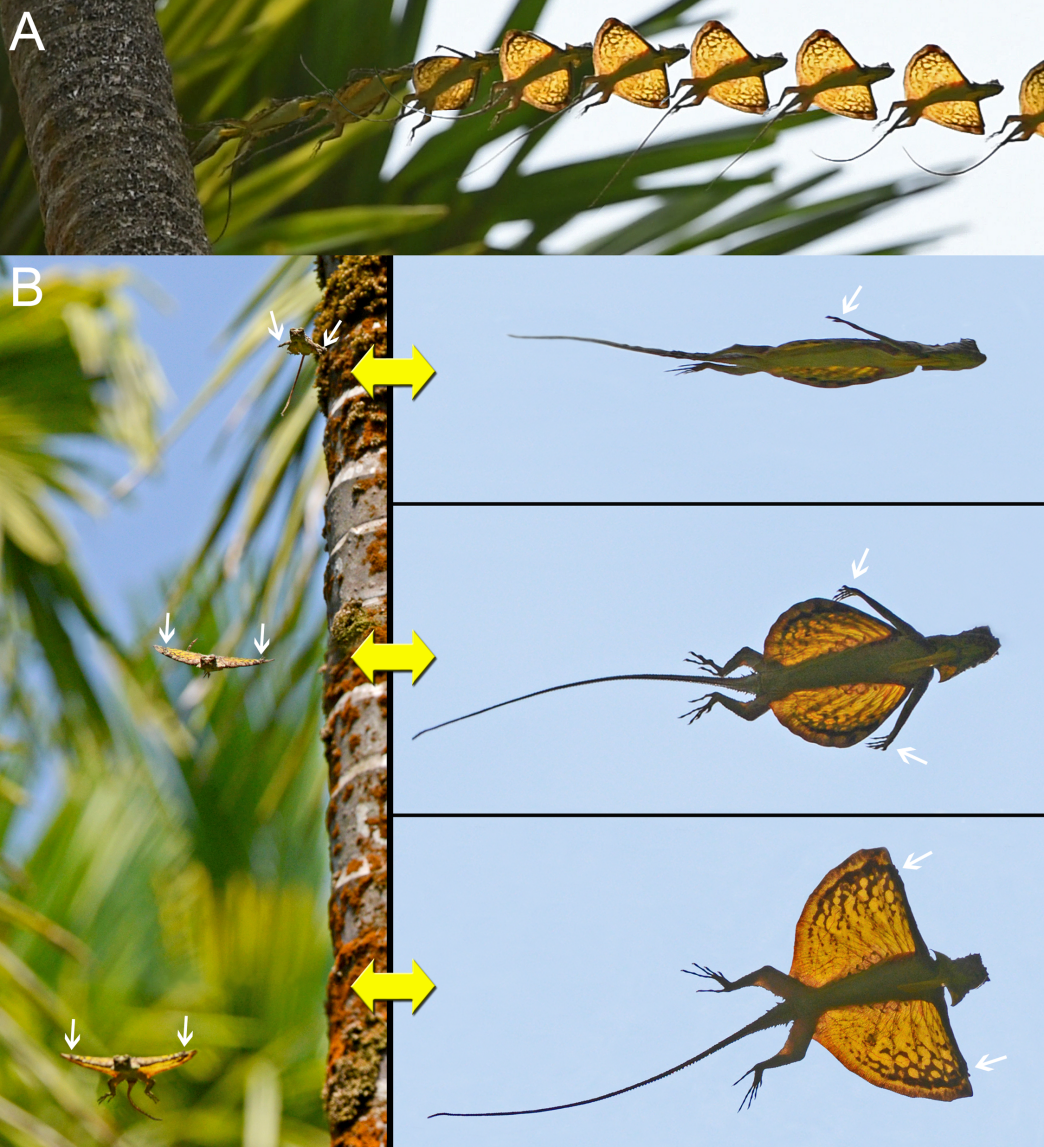
Formation of the composite wing of *Draco dussumieri* during the initial phases of the gliding flight. (A) Seen from below. Image composed of ten still frames from a video clip (S1 Video); consecutive frames are 1/30 s apart from each other. (B) Seen from the front (left, consecutive photos are 181 ms apart from each other) and from below (right; corresponding photos of the same phases). White arrows indicate the position of the hands. For explanation see text.

**Fig 2:**
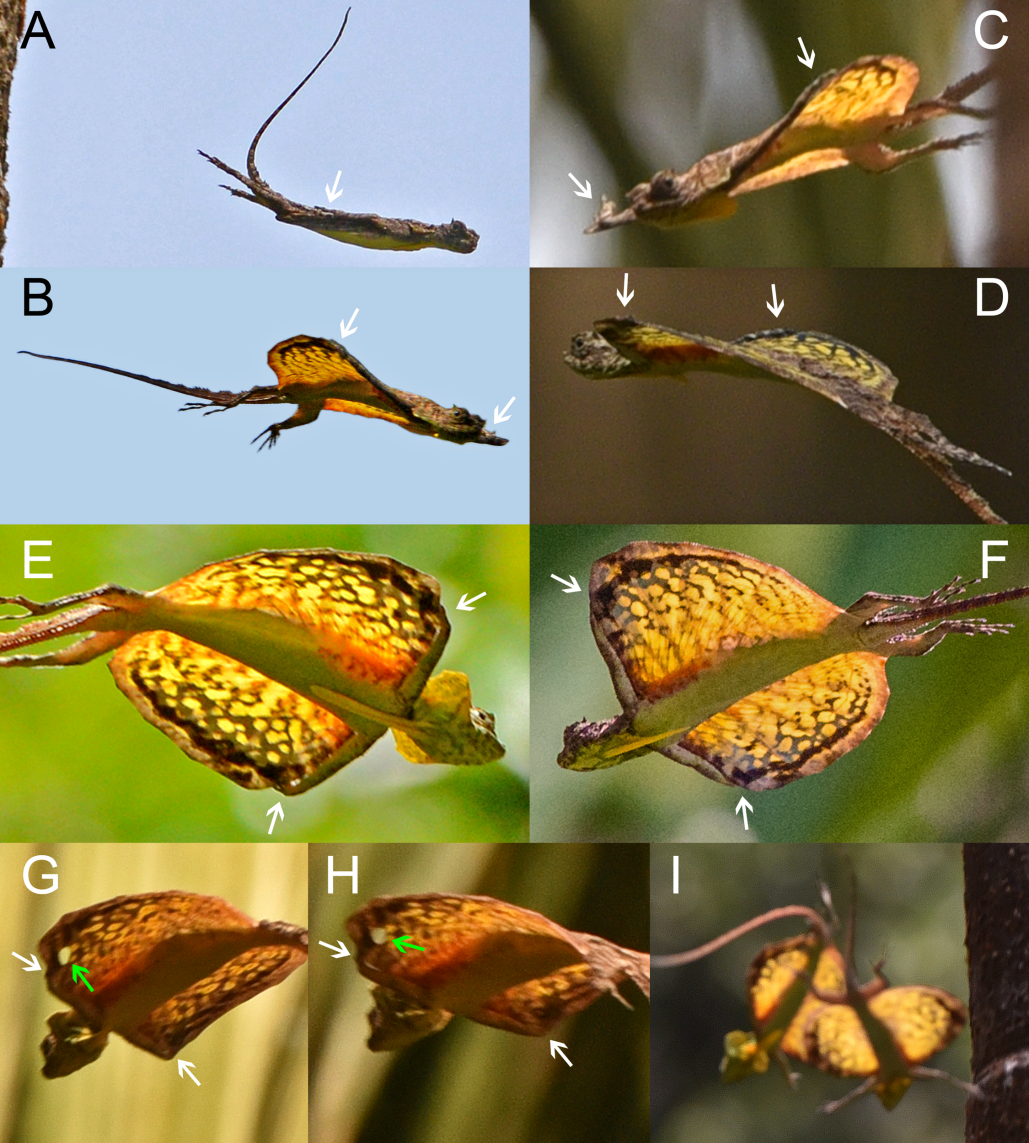
Details of the composite wing in *Draco dussumieri*. White arrows indicate the position of the hands. (A) During takeoff jump. Note the straight back while the patagium is furled. (B) During glide phase. Note the cambered shape of the patagium and the arching of the back when the patagium is extended. (C- F) During glide phase, seen from the side (C, D) and from below (E, F). Note the strongly adducted hands and the fingers touching the dorsal side of the patagium. The postaxial part of the upper arm is below the leading edge of the patagium. (G, H) Apparent rupture of the patagium (green arrow) posterior to second elongated rib. (I) Two males uncontrolledly descending during agonistic interaction without their forelimbs being in contact with the not fully extended patagium.

Sequential photographs and videos of gliding *Draco* revealed that the lizards did not hold their forelimbs free and extended forwardly in front of the body while airborne but instead always attached them to the leading edge of the patagium (Figs 1 and 2; S1 Fig). This combination constitutes a hitherto unknown type of composite wing, in which the separate units are formed by different parts of the body and are connected to each other only for the duration of the gliding flight.

### Formation of the composite wing

**Fig 3:**
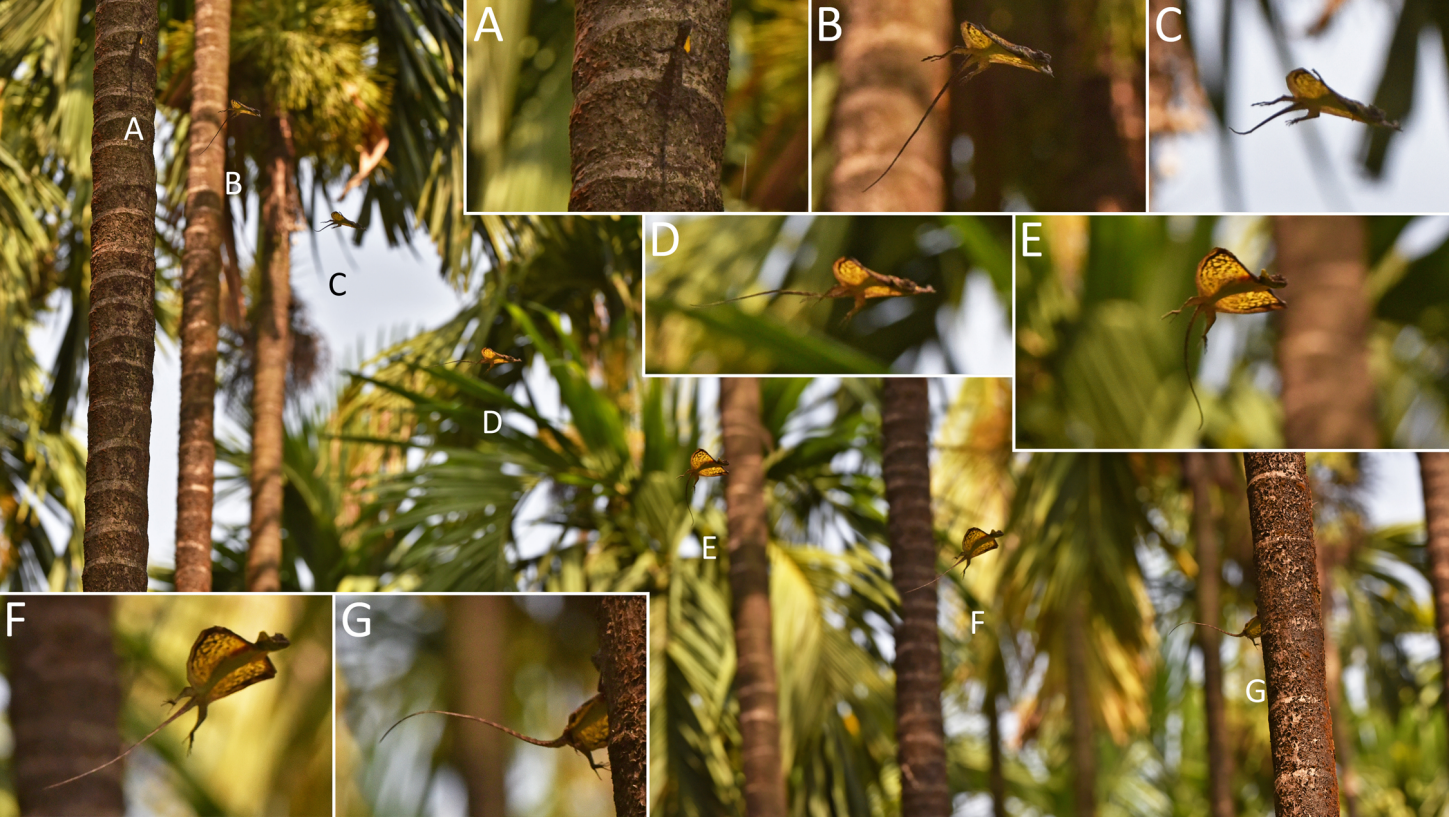
*Draco dussumieri* (adult male) gliding from one tree to another. Sequential photographs were taken at 6.5 frames/s. (A) Starting point where the lizard had been perching and displaying. (B) After the initial jump, the patagium is spread and the hands are moved above the patagium. (C) The hands are being placed on the dorsal side of the leading edge of the patagium. (D) After the composite wing is formed, the glide path becomes more horizontal. (E- F) Before landing, the glide direction is adjusted and the patagium is increasingly oriented upward, accompanied by a strong upturn of the glide path. (G) During landing, the forelimbs are extended forward towards the landing spot, and the head is moved up and back.

At the beginning of a gliding flight, the movements and actions of the forelimbs and the patagium followed a particular pattern in all observed and documented instances. Initially, a lizard oriented its body horizontally on the tree trunk, then launched itself from the tree by jumping and descended head first (Figs 1–3; S2 Fig). After takeoff, it reoriented its body, so that the ventral side was directed towards the ground. The extended forelimbs reached back and up, and the anterior patagium-supporting ribs were spread (Figs 1 and 3C). When the extended arms were parallel to the leading edge of the patagium and the strongly adducted hands were above the anterior two patagial ribs, the hands were placed on the dorsal surface of the outer margins of the leading edge of the patagium. Often, the hands were not placed on both sides simultaneously but on one shortly after the other, causing a faint swerve in the glide path (S2 Animation). The resulting composite wing was subsequently extended a little further forward to its full extent (Figs 1–3). The whole process is shown in Figs 1 and 3; S2 and S3 Figs; S1–S3 Videos (original speed); and S1–S3 Animations (slowed down tenfold).

During and after the spreading of the patagium, the lizards actively arched their backs, and the extended patagium was cambered (Fig 2A and 2B). The attached forelimbs constituted a straight, thick leading edge of the aerofoil, which contrasted with its thin trailing edge (Figs 1–3). While being in contact with the patagium, the hands were deviated about 90° ulnarly relative to the extended arms (Figs 1 and 2).

When the composite wing was formed, the postaxial parts of the upper arms were situated along the ventral side of the leading edge of the patagium whereas the lower arm reached slightly dorsally, allowing the adducted hands to touch the dorsal side of the patagium (Fig 2). One specimen exhibited an apparent rupture of the patagial membrane (Fig 2G and 2H).

The timing of the formation of the composite wing varied between different glides. From takeoff (feet leaving the tree trunk) to the completion of the formation between 167 ms (Fig 1; S1 Video; S1 Animation) and about 460 ms (Fig 3; S3 Fig) passed. During an agonistic interaction, two rivalling males fell down a tree after attacking each other. They spread their patagia but did not attach their forelimbs to them but instead extended the arms towards their opponents and the tree trunk while airborne (Fig 2I). Both specimens tumbled down along the tree trunk and their descent was not directed. One lizard finally grasped the bark of the tree about one meter above the ground; the second specimen hit the ground.

### Glide trajectory

The glide trajectories varied markedly between individual glides, most notably in the horizontal distance between the starting and the landing point. The longest observed glides covered a horizontal distance of about 26 m, the shortest only 2.2 m to the next tree. In two instances, the glide ended several meters below the starting point on the same tree, when one lizard glided in one complete spiral downwards around the tree trunk and a second lizard, on its way away from the tree, turned almost 180° in mid-air and glided back downwards to the tree from which it had started.

**Fig 4:**
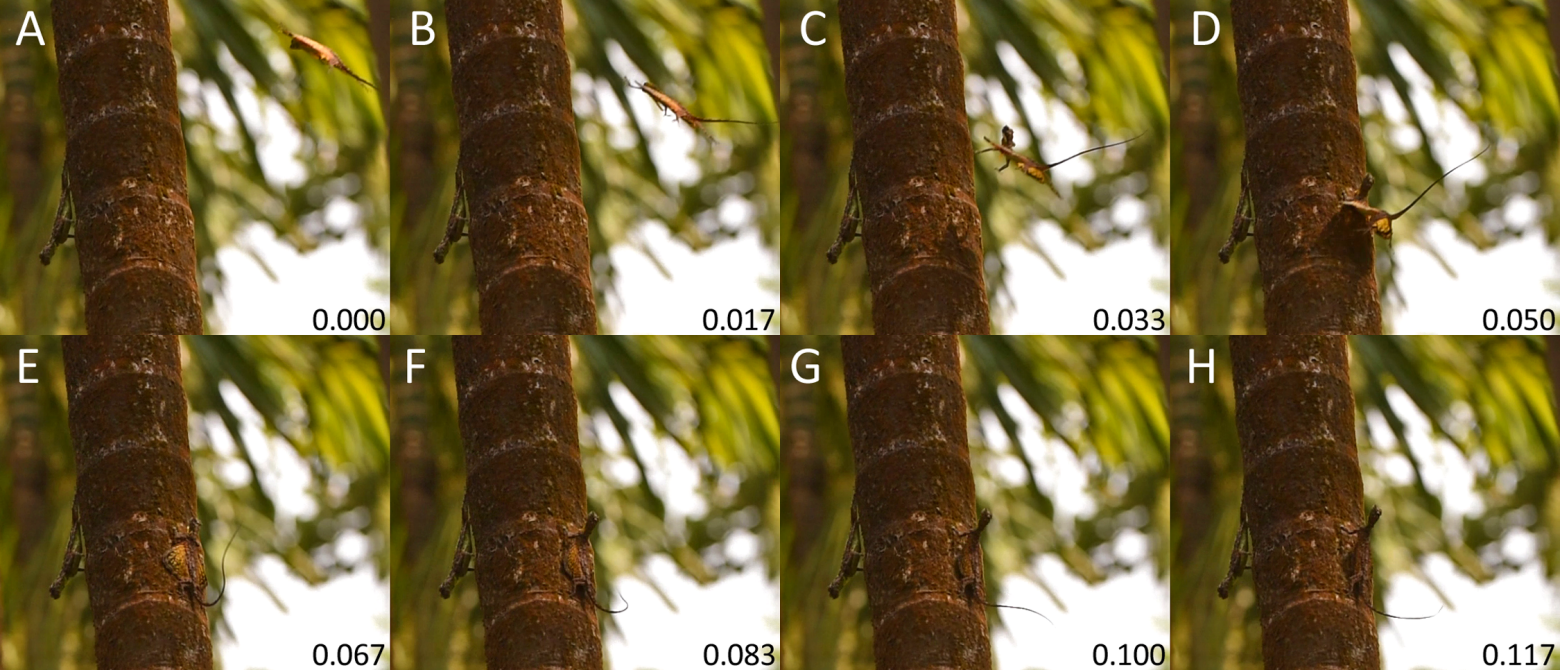
Landing of *Draco dussumieri* at the end of a gliding flight. Still frames from a video sequence (S4 Animation); consecutive images are 1/60 s apart from each other. (A) The lizard approaches the landing site and its hands are lifted from the surface of the patagium. (B) The hands are free and the arms move forward towards the landing site. (C) Immediately before the impact, the forelimbs are extended forward and the head is moved up and back. The furling of the patagium begins. (D) Arms and chest but not the head hit the landing site. The hands grasp the surface. (E) Legs, abdomen and head hit the landing site. (F-H) The body is erected and the furling of the patagium is completed.

**Fig 5:**
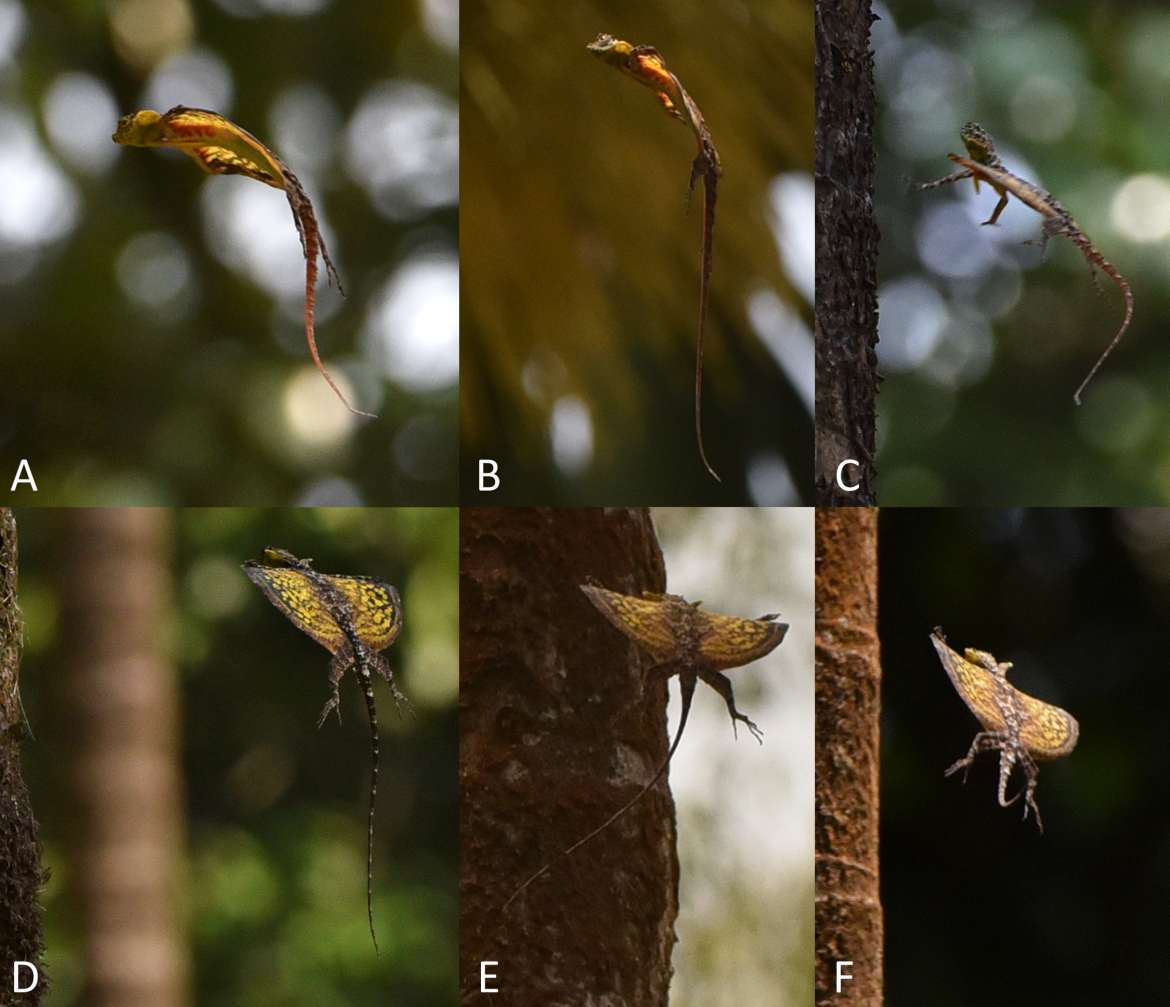
*Draco dussumieri* disconnecting the composite wing immediately before landing. Photographs A and B show the same individual on two different instances, C a second individual, and D-F a third individual at three different instances. (A) Shortly before reaching the landing point, the aerofoil is orientated more vertically. The fingers are still touching the leading edge of the patagium. Note the ulnar deviation of the hands. (B) The hands have just been lifted from the patagial surface and are still strongly adducted. (C) At a remaining distance of only a few centimeters from the landing point, the forelimbs and hindlimbs are extended forward towards the tree. (D-F) Different states during the release of the patagium: (D) the fingers of the left hand have been lifted up and thus have just lost contact with the patagium; the fingers of the right hand still touch the leading edge. (E) the fingers of the left hand have just been lifted from the dorsal surface of the patagium; the right hand has already been rotated forward. (F) the left hand is rotated forward; the right forelimb is already moved forward and not visible from behind anymore.

A gliding flight typically consisted of four phases: the initial takeoff jump, an acceleration phase, a gliding phase, and the landing. The relative duration of the different phases varied between individual gliding flights. During the initial takeoff jump, the lizard left the tree with a forward speed of approximately 2 m*s^-1^ (S2 Fig). Once airborne, it was rapidly accelerated downwards in addition to the continued forward motion (Fig 3; S3 Fig, S2 Video). The acceleration phase ended with the formation of the composite wing, after which the trajectory became more horizontal (Figs 1B and 3D; S3 Fig). In two documented glides over a horizontal distance of 8 and 12 m, respectively, the lizards reached their maximum velocity of about 6.8 m*s^-1^ (5.8 m*s^-1^ horizontal speed) in this phase of the glide (S3 Fig). Shortly before reaching the landing point, the hands with the lateral parts of the leading edge of the patagium were raised above the horizontal body plane (Fig 3; S3 and S4 Figs). The aerofoil was re-orientated upward, and the trajectory subsequently became more horizontal or even turned upwards (Fig 3; S3 and S4 Figs). The longitudinal axis of the lizard’s body and the aerofoil were finally orientated perpendicular to the glide path, and the velocity of the forward motion was drastically reduced (Figs 3–5; S3 and S4 Figs). This upward orientation of the body was retained until the landing point was reached (Figs 3–5; S3 and S4 Figs; S4 Animation). The forelimbs remained attached to the patagium during the gliding phase and most of the landing phase (Fig 3; S3 and S4 Figs). Immediately before landing, the hands were lifted from the patagium and the forelimbs were moved toward the landing spot to allow the hands to grasp the surface being landed upon (Figs 3–5; S3 and S4 Figs; S4 Animation). During the landing process, the patagium was being furled against the sides of the body (Figs 3G, 4, and 5C; S4 Animation). In most of the observed gliding flights, the trajectory continued to be orientated downward during the landing, albeit markedly more horizontal than during the preceding glide phases, and the lizards hit the surface first with the forelimbs then with the hindlimbs (Figs 3G, 4D, and 5C; S4 Animation). At the end of longer glides, the trajectory often became horizontal or even turned upward and the lizards hit the surface with forelimbs and hindlimbs almost simultaneously (S3 and S4 Figs).

### Disconnecting the composite wing

The composite wing was disconnected immediately before reaching the landing point to enable the hands to grasp the surface (Figs 3–5; S3 and S4 Figs; S4 Animation). The whole process, from the fingers leaving the surface of the patagium until the forelimbs being extended forward, was completed within only about 1/30 s (Fig 4), and was therefore difficult to resolve temporally in detail with the equipment used in this study. Several photographs taken at different stages of the disconnection process indicate that the fingers are uplifted while the palm of the hand remains on the dorsal surface of the patagium; the hands are then rotated toward the longitudinal axis of the arm, reversing the adduction; and finally, the whole forelimbs are moved forward until they are orientated towards the landing point (Figs 4 and 5).

## Discussion

My findings demonstrate that, contrary to previous assumptions, the forelimbs of *Draco* are not held free and extended forwardly during flight but are always attached to the patagium. The involvement of the forelimbs in the formation of such a hitherto unknown composite wing suggests a plausible explanation of how the lizards achieve manoeuvrability while airborne, in that the forelimbs are connected to the leading edge of the patagium and control the aerofoil during the gliding flight.

The patagium of *Draco* lizards can be spread by the intercostal musculature without the help of the forelimbs, as is evident during display and during the formation of the composite wing. However, given the limited number of muscles inserting the patagium-supporting ribs [7] and the resulting constraints regarding the directions of movements that can be produced by these muscles, it appears unlikely that the trunk muscles are the major controlling unit of the wing. In contrast to that, the forelimbs can be moved in a much wider range of directions and, when attached to the patagium, appear to be well suited to perform the fine-scale alterations of shape and orientation of the aerofoil through adjustments of the leading edge, which are necessary for manoeuvring and controlling the trajectory. Although my observations suggest that *Draco* controls its wing with the forelimbs, it remains to be tested experimentally whether the lizards would still be able to conduct directed glides if the forelimbs were manipulatively prevented from forming a connection with the patagium.

A number of morphological characteristics support the notion that the forelimbs are connected to the patagium. *Draco* is able to deviate its hand ulnarly, but not markedly radially, whereas other arboreal agamid lizards can neither adduct nor abduct their hands at the wrist joint (S1 Table). Therefore, the ability to adduct the hand is obviously not directly related to climbing activities and appears to be a specific adaptation that allows the grasping of the patagium by the hand. The pronounced adduction of the wrist brings the finger claws in a position that is best suited to exert forward traction on enlarged scale rows that extend along the first two pairs of ribs on the dorsal surface of the patagium [8]. The relative length of the fingers of *Draco* differs from that of other arboreal agamid lizards, inasmuch as the third and fourth fingers (the longest) are almost identical in length, and the somewhat shorter second and fifth fingers likewise have almost the same length (S1 Table). This appears to be an adaptation that enables the fingers to grasp modified areas above the ribs on the patagial surface. The claws of the two longest fingers reach to enlarged scales posterior to the second elongated rib, the shorter ones reach between the first and the second rib when the forelimb is placed along the leading edge of the patagium and the wrist is adducted. A live specimen of *Draco dussumieri* (Fig 3G and 3H) and two preserved specimens (ZFMK-H 14098: *Draco dussumieri*; ZFMK 20897: *Draco volans*; S1 Table) exhibited apparent ruptures of the patagial membrane just behind the second elongated rib, which is the area where the claws of the longer fingers touch the patagium.

To grasp the patagium with the hands, opposing forces would need to be exerted onto the two sides of the leading edge. When the composite wing was formed, the postaxial part of the upper arm was below the leading edge of the patagium whereas the adducted hands were on the dorsal side (Figs 1 and 2; S1 Fig). Thus, the leading edge of the patagium appeared to be locked between the arms pushing up from the ventral side and the hands applying downward pressure and forward traction on the dorsal side (Fig 2). Details of how the connection of the forelimb to the leading edge is achieved and what forces are produced by the respective body parts involved need to be further investigated.

As *Draco* shows a conserved morphology of the patagium within the genus [6], and the extended forelimb constantly reaches close to the lateral margin of the leading edge (S1 Table), the need for control of the patagium through the forelimbs could be an important constraint preventing further rib elongation and increase of wing area. The wing of *Draco* is characterized by distinct adaptive morphological features. According to aerodynamic theory, the camber of the aerofoil and the presence of a thick leading edge, compared to the thin trailing edge, may create greater lift forces than is possible using flat wings [3,19]. If this is the case in the comparatively short and broad wings of *Draco* remains to be tested experimentally. Examination of preserved *Draco* specimens suggest that the cambering and the tension of the patagium can be altered by arching or straightening of the back and, to a lesser extent, movements of the hindlimbs, as the posterior end of the patagium is attached to the proximal portions of the thighs. These adjustments certainly have an influence on the aerodynamic properties of the aerofoil, but their significance remains to be studied in detail. Also, the role of the throat lappets which are extended laterally in some but not all gliding flights (Figs 1–5; S1–S3 Fig) and which have been interpreted as canards [20] or smaller and secondary anterior aerofoils [6] remains to be investigated in detail.

**Fig 6:**
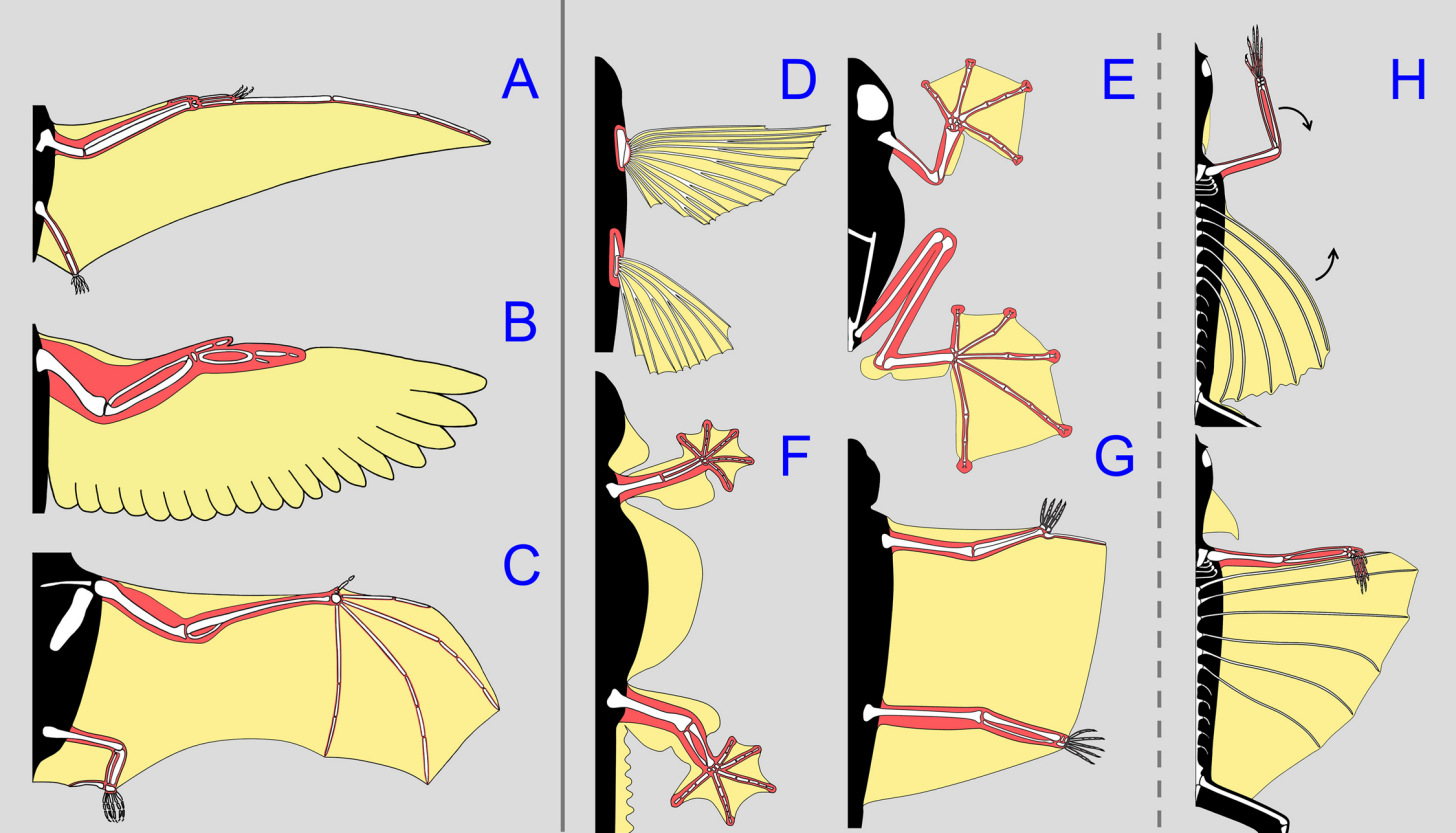
**Wings and patagia of vertebrate groups employing flapping (A-C) and gliding (D-H) flight.** Colours mark the major aerodynamic surfaces (yellow) and the skeletal and muscular structures that control them (red). A: Pterosaur (*Rhamphorhynchus*, extinct); B: Bird (*Columba*); C: Bat (*Phyllostomus*); D: Flying fish (*Hirundichthys*); E: Flying frog (*Rhacophorus*); F: Parachuting gecko (*Ptychozoon*); G: Flying squirrel (*Petaurista*); H: Flying lizard (*Draco*). In *Draco*, the forelimbs are connected to the lift-generating surfaces of the patagium only for the duration of the flight.

A composite wing, in which the lift-generating and the controlling units are formed independently by different parts of the body and must be connected to each other at the beginning of the gliding flight, is hitherto unknown from the animal kingdom (Figs 1–3 and 6). Apart from few groups of gliding or parachuting animals that use only their flattened bodies and unmodified, outstretched limbs to generate lift and drag forces [2, 21,22], all other groups of flying and gliding animals have developed enlarged aerodynamic surfaces that are permanently attached to the skeletal and muscular elements that control them [2,3,19]. The enlarged aerodynamic surfaces of vertebrates are mostly found on modified limbs or fins (Fig 6). In contrast to the hindlimbs, which possess moderate modifications, such as a lateral compression and a row of enlarged scales along the trailing edge of the thigh [6,8], the forelimbs of *Draco* lack modifications that would increase their surface. Such modifications would be expected if the forelimbs were held free and extended forwardly and used to generate greater drag and lift forces, as in parachuting geckos and frogs [23,24] (Fig 6). The major advantage of the composite wing of *Draco* is that the forelimbs retain their complete range of movement and full functionality for agile climbing and running activities when not being used as a part of the wing. Although this study reports only on the formation of the composite wing in *D*. *dussumieri*, this behaviour is evident in previously published photographs of gliding specimens of other *Draco* species [2,5,25]. Given the conserved patagial and forelimb morphology across all species of *Draco* [6] (S1 Table), the composite wing is very likely formed in the same way by all species in the genus.

The patagium of *Draco* differs functionally from the patagia of the parachuting geckos *Ptychozoon* and *Hemidactylus* (Fig 6F), as the latter are unsupported by ribs, not controlled by muscles, and unfold passively as they catch air during descent [8]. The composite wing of *Draco* closely resembles the plagiopatagia of gliding squirrels and colugos, which extend between the arms and legs and are controlled by limb movements [2] (Fig 6G). The patagium of *Draco*, however, is not spread as a result of the limbs posturing while airborne, but apparently has to be deliberately grasped and thus connected to the forelimbs.

The estimated maximum glide velocity of approximately 6.8 m*s^-1^ is within the range of adjusted mean glide velocity measured under experimental conditions for 11 other species of *Draco* (5.2–7.6 m*s^-1^) [5]. Further measurements will certainly reveal a wider range of glide velocities than that obtained from my limited number of documentations that allowed an analysis of that kind.

The concept of a patagium being controlled by largely unmodified limbs needs to be taken into consideration when interpreting extinct organisms as possible gliders. A number of extinct lineages, including the Late Permian *Coelurosauravus*, the Late Triassic *Kuehneosaurus*, *Kuehneosuchus* and *Icarosaurus*, the Late Triassic *Mecistotrachelos*, and the Early Cretaceous *Xianglong*, possess elongated ribs or bony rib-like structures that are hypothesized to have supported a patagial membrane and thus to resemble the glide-associated morphological modifications of the modern *Draco* [20,26–30]. These fossil taxa are assumed to have glided through the air with the forelimbs extended forwardly and to have changed direction by unilateral adjustments of the aerofoil through contractions of the trunk musculature [6,20,27,28]. Since *Draco* apparently uses the forelimbs to control the patagium, it is reasonable to propose that the extinct gliders formed a similar connection and might have regulated their glide path in a similar way. Skeletal properties of extinct gliders allow this interpretation. The forelimb is shorter than the first elongated rib in these species and would have constituted a straight, thickened leading edge when extended and attached to the patagium. To hold on to the dorsal surface of the patagium, adduction of the wrist is advantageous, a condition which is apparent in the holotypes of *Icarosaurus siefkeri* and *Mecistotrachelos apeoros* and in a well-preserved specimen of *Coelurosauravus jaekeli* [28,29,31]. Hence, it seems plausible that these early reptile gliders likewise formed a composite wing using their forelimbs. This would imply that the manner of how modern *Draco* form their wing and apparently control an aerofoil while simultaneously retaining full mobility of the forelimb could have been developed convergently in the past by several non-related reptile lineages.

## Supporting information

S1 Table Results of the morphological examination of voucher specimens of arboreal agamid lizards. For details and abbreviations see Materials and Methods. Symbols indicate the ability to abduct/adduct the wrist more than 80° (*) or less than 20° (–).
(DOCX)

S1 Fig Composite image showing the connection of the forelimb to the leading edge of the patagium in airborne *Draco dussumieri* in 108 different instances (gliding flights). (TIF)

S2 Fig *Draco dussumieri* forming the composite wing during the initial phases of a gliding flight. Consecutive still frames from a video (S2 Video) recorded at 60 frames/s. Numbers in the upper right corner are the running time given in seconds.
(TIF)

S3 Fig Gliding flight of a male *Draco dussumieri*. Composite image composed of photos taken at a rate of 6.5/s, showing the different glide phases, and details of the corresponding individual photos. The lizard jumps from a tree (upper right corner) and forms its composite wing. Subsequently, the trajectory becomes more horizontal. Before landing, the aerofoil is orientated upwards and the forward speed is reduced. The lizard approaches the landing point in a horizontal trajectory, hitting it with hands and feet almost simultaneously.
(TIF)

S4 Fig Landing of *Draco dussumieri* at the end of a gliding flight covering a horizontal distance of 12 m. Hands with the lateral parts of the leading edge are raised above the horizontal body plane, causing a change of the angle of attack (right). The trajectory first becomes horizontally than turns even upwards. Eventually, the aerofoil is brought in a near vertical position and the forward speed is reduced. Note the forelimbs being connected to the leading edge of the patagium (right and middle).
(TIF)

S1 Video Formation of the composite wing in *Draco dussumieri* during the initial phases of a gliding flight. Video recorded at 60 frames/s, original speed. See also S1 Animation (slowed down tenfold) and Fig 1.
(WMV)

S2 Video Formation of the composite wing in *Draco dussumieri* during the initial phases of a gliding flight. Video recorded at 60 frames/s, original speed. See also S2 Animation (slowed down tenfold).
(WMV)

S3 Video Formation of the composite wing in *Draco dussumieri* during the initial phases of a gliding flight. Video recorded at 60 frames/s, original speed. See also S3 Animation (slowed down tenfold).
(WMV)

S1 Animation Formation of the composite wing in *Draco dussumieri* during the initial phases of a gliding flight. Animation in “Graphics Interchange Format” (GIF) using the still frames from S1 Video slowed down tenfold. See also Fig 1.
(GIF)

S2 Animation Formation of the composite wing in *Draco dussumieri* during the initial phases of a gliding flight. Animation in GIF using the still frames from S1 Fig (S2 Video) slowed down tenfold.
(GIF)

S3 Animation Formation of the composite wing in *Draco dussumieri* during the initial phases of a gliding flight. Animation in GIF using the still frames from S3 Video slowed down tenfold.
(GIF)

S4 Animation Landing of *Draco dussumieri* at the end of a gliding flight. Animation in GIF using the still frames from a video recorded at 60 frames/s, slowed down twentyfold. See also Fig 4.
(GIF)

## References

[1] Dudley R, Byrnes G, Yanoviak SP, Borrell B, Brown RM, McGuire JA. Gliding and the functional origins of flight: biomechanical novelty or necessity? Annu Rev Ecol Evol Syst. 2007; 38: 179–201.

[2] Socha JJ, Jafari F, Munk Y, Byrnes G. How animals glide: from trajectory to morphology. Can J Zool. 2015; 93: 901–924.

[3] Lingham-Soliar T. The vertebrate integument, volume 2: Structure, design and function Berlin & Heidelberg: Springer; 2015.

[4] McGuire JA. Allometric prediction of locomotor performance: an example from Southeast Asian flying lizards. Am Nat. 2003; 161: 337–349. doi: 10.1086/346085 12675377 10.1086/346085

[5] McGuire JA, Dudley R. The cost of living large: comparative gliding performance in flying lizards (Agamidae: *Draco*). Am Nat. 2005; 166: 93–106. doi: 10.1086/430725 15937792 10.1086/430725

[6] McGuire JA, Dudley R. The biology of gliding in flying lizards (genus *Draco*) and their fossil and extant analogs. Integr. Comp Biol. 2011; 51: 983–990. doi: 10.1093/icb/icr090 21798987 10.1093/icb/icr090

[7] Colbert EH. Adaptations for gliding in the lizard *Draco*. Am Mus Novit. 1967; 2283: 1–20.

[8] Russell AP, Dijkstra LD. Patagial morphology of *Draco volans* (Reptilia: Agamidae) and the origin of glissant locomotion in flying dragons. J Zool. 2001; 253: 457–471.

[9] Seba A. Locupletissimi rerum naturalium thesauri accurata descriptio, et iconibus artificiosissimis expressio, per universam physices historiam Cui, in hoc rerum genere, nullum par exstitit. Ex toto terrarum orbe collegit, digessit, descripsit, et depingendum curavit. Tomus I. Amstelaedami: J. Wetstenium, & Gul. Smith, & Janssonio-Waesbergios; 1734.

[10] Marsden W. The history of Sumatra, containing an account of the government, laws, customs and manners of the native inhabitants Third edition London: The author, J. M'Creery, Black-Court and Longman, Hurst, Rees, Orme, and Brown, Paternoster-Row; 1811.

[11] Günther ACLG. On the reptiles and amphibians of Borneo. Proc Zool Soc Lond. 1872; 1872: 586–600.

[12] Maindron MM. Dragons, fabled and real. Pop Sci Monthly. 1890; 36: 808–813.

[13] John KO. On the ‘patagial musculature’ of the South Indian flying lizard *Draco dussumieri*, Dum & Bib. Brit J Herpetol. 1970; 4: 161–168.

[14] Ratnovsky A, Elad D, Halpern P. Mechanics of respiratory muscles. Respir Physiol Neurobiol. 2008; 163: 82–89. doi: 10.1016/j.resp.2008.04.019 18583200 10.1016/j.resp.2008.04.019

[15] Hairstone NG. Observations on the behavior of *Draco volans* in the Philippines. Copeia. 1957; 1957: 262–265.

[16] John KO. Observations on the mating behaviour and copulation in *Draco dussumieri* Dum. & Bib. (Reptilia: Sauria). J Bomb Nat Hist Soc. 1967; 64: 112–115.

[17] Mori A, Hikida T. Natural history observations of the flying lizard, *Draco volans sumatranus* (Agamidae, Squamata) from Sarawak, Malaysia. Raffles B Zool. 1993; 41: 83–94.

[18] Manthey U. Agamid lizards of southern Asia. / Agamen des südlichen Asien. Draconinae 1 (Terralog 7a). Frankfurt am Main: Edition Chimaira; 2008.

[19] Norberg UM. Vertebrate Flight. Berlin: Springer; 1990.

[20] Stein K, Palmer C, Gill PG, Benton MJ. The aerodynamics of the British late Triassic Kuehneosauridae. Palaeontology. 2008; 51: 967–981.

[21] Vanhooydonck B, Meulepas G, Herrel A, Boistel R, Tafforeau P, Fernandez V, et al Ecomorphological analysis of aerial performance in a non-specialized lacertid lizard, *Holaspis guentheri*. J Exp Biol. 2009; 212: 2475–2482. doi: 10.1242/jeb.031856 19617441 10.1242/jeb.031856

[22] Yanoviak SP, Dudley R, Kaspari M. Directed aerial descent in canopy ants. Nature. 2005; 433: 624–626. doi: 10.1038/nature03254 15703745 10.1038/nature03254

[23] Emerson SB, Koehl MAR. The interaction of behavioral and morphological change in the evolution of a novel locomotor type: “flying” frogs. Evolution. 1990; 44: 1931–1946. doi: 10.1111/j.1558-5646.1990.tb04300.x 28564439 10.1111/j.1558-5646.1990.tb04300.x

[24] Young BA, Lee CE, Daley KM. On a flap and a foot: aerial locomotion in the “Flying” Gecko, *Ptychozoon kuhli*. J Herpetol. 2002; 36: 412–418.

[25] Lee C. On the wings of dragons. 2015. Available from http://www.wildborneo.com.my/blog/.

[26] Robinson PL. Gliding lizards of the Upper Keuper of Great Britain. Proc Geol Soc Lond. 1962; 1601: 137–146.

[27] Colbert EH. The Triassic gliding reptile *Icarosaurus*. Bull Amer Mus Nat Hist. 1970; 143: 85–142.

[28] Frey E, Sues HD, Munk W. Gliding mechanism in the Late Permian reptile *Coelurosauravus*. Science. 1997; 275: 1450–1452.

[29] Fraser NC, Olsen PE, Dooley AC, Ryan TR. A new gliding tetrapod (Diapsida:? Archosauromorpha) from the upper Triassic (Carnian) of Virginia. J Vert Paleontol. 2007; 27: 261–265.

[30] Li PP, Gao KQ, Hou LH, Xu X. A gliding lizard from the early Cretaceous of China. Proc Natl Acad Sci U.S.A. 2007; 104: 5507–5509. doi: 10.1073/pnas.0609552104 17376871 10.1073/pnas.0609552104 PMC1838464

[31] Colbert EH. A gliding reptile from the Triassic of New Jersey. Am Mus Novit. 1966; 2246: 1–23.

