# Supplementary material for: How lizards fly: A novel type of wing in animals
Source: PLoS One. 2017 Dec 13;12(12):e0189573. doi: 10.1371/journal.pone.0189573 (PMC5728497; doi:10.1371/journal.pone.0189573)
Supplement: S1 Table — For details and abbreviations see Materials and Methods. Symbols indicate the ability to abduct/adduct the wrist more than 80° (*) or less than 20° (–). (DOCX) [file pone.0189573.s001.docx]

Online supporting information

Table S1. Results of the morphological examination of voucher specimens of arboreal agamid lizards. For details and abbreviations see Materials and Methods. Symbols indicate the ability to deviate the wrist more than 80° (*) or less than 20° (–). Numbers for “finger length” indicate Finger IV > Finger III/Finger V > Finger II (0); or Finger III = Finger IV (1), and Finger II = Finger V (2).

|  |  | SVL | AL | LL | deviation of wrist | | finger length |
| --- | --- | --- | --- | --- | --- | --- | --- |
| specimen | species | [mm] | [mm] | [% of AL] | ulnarly | radially |  |
| ZFMK 16630 | *Draco blanfordii* | 96.3 | 31.6 | 118 | * | – | 1, 2 |
| ZFMK 44018 | *Draco blanfordii* | 130.1 | 43.7 | 113 | * | – | 1, 2 |
| ZFMK 44021 | *Draco blanfordii* | 101.2 | 34.3 | 114 | * | – | 1, 2 |
| ZFMK-H 14098 | *Draco dussumieri* | 65.6 | 20.1 | 130 | * | – | 1, 2 |
| ZFMK-H 14099 | *Draco dussumieri* | 68.0 | 22.7 | 134 | * | – | 1, 2 |
| ZFMK-H 14100 | *Draco dussumieri* | 68.0 | 22.5 | 132 | * | – | 1, 2 |
| ZFMK 51780 | *Draco fimbriatus* | 30.5 | 7.7 | 121 | * | – | 1, 2 |
| ZFMK 16492 | *Draco fimbriatus* | 97.6 | 28.7 | 136 | * | – | 1, 2 |
| ZFMK 43698 | *Draco fimbriatus* | 115.7 | 34.1 | 137 | * | – | 1, 2 |
| ZFMK 43699 | *Draco fimbriatus* | 118.4 | 36.9 | 128 | * | – | 1, 2 |
| ZFMK 43700 | *Draco fimbriatus* | 107.6 | 38.6 | 117 | * | – | 1, 2 |
| ZFMK 43701 | *Draco fimbriatus* | 110.8 | 35.6 | 114 | * | – | 1, 2 |
| ZFMK 88943 | *Draco indochinensis* | 84.3 | 27.8 | 123 | * | – | 1, 2 |
| ZFMK 88944 | *Draco indochinensis* | 119.8 | 40.3 | 118 | * | – | 1, 2 |
| ZFMK 88945 | *Draco indochinensis* | 107.2 | 32.8 | 120 | * | – | 1, 2 |
| ZFMK 20898 | *Draco lineatus* | 75.7 | 25.9 | 115 | * | – | 1, 2 |
| ZFMK 88938 | *Draco maculatus* | 69.8 | 19.9 | 127 | * | – | 1, 2 |
| ZFMK 88939 | *Draco maculatus* | 63.2 | 19.0 | 137 | * | – | 1, 2 |
| ZFMK 88940 | *Draco maculatus* | 66.4 | 19.1 | 133 | * | – | 1, 2 |
| ZFMK 88941 | *Draco maculatus* | 63.3 | 18.3 | 127 | * | – | 1, 2 |
| ZFMK 50522 | *Draco melanopogon* | 81.5 | 28.4 | 125 | * | – | 1, 2 |
| ZFMK 50523 | *Draco melanopogon* | 74.6 | 25.8 | 129 | * | – | 1, 2 |
| ZFMK 50526 | *Draco maximus* | 127.5 | 39.0 | 117 | * | – | 1, 2 |
| ZFMK 50524 | *Draco obscurus* | 79.8 | 27.1 | 120 | * | – | 1, 2 |
| ZFMK 50525 | *Draco obscurus* | 78.8 | 25.2 | 118 | * | – | 1, 2 |
| ZFMK 65773 | *Draco* cf. *obscurus* | 79.0 | 28.6 | 129 | * | – | 1, 2 |
| ZFMK 50520 | *Draco quinquefasciatus* | 81.8 | 26.6 | 132 | * | – | 1, 2 |
| ZFMK 50521 | *Draco quinquefasciatus* | 88.7 | 27.0 | 135 | * | – | 1, 2 |
| ZFMK 84900 | *Draco quinquefasciatus* | 87.5 | 26.1 | 128 | * | – | 1, 2 |
| ZFMK 63613 | *Draco ornatus* | 79.5 | 25.8 | 118 | * | – | 1, 2 |
| ZFMK 20900 | *Draco reticulatus* | 76.5 | 24.3 | 122 | * | – | 1, 2 |
| ZFMK 57811 | *Draco spilopterus* | 66.3 | 24.2 | 126 | * | – | 1, 2 |
| ZFMK 84303 | *Draco spilopterus* | 68.2 | 22.1 | 130 | * | – | 1, 2 |
| ZFMK 13369 | *Draco sumatranus* | 68.5 | 19.9 | 138 | * | – | 1, 2 |
| ZFMK 13370 | *Draco sumatranus* | 65.7 | 18.3 | 136 | * | – | 1, 2 |
| ZFMK 13170 | *Draco taeniopterus* | 74.7 | 26.2 | 138 | * | – | 1, 2 |
| ZFMK 16631 | *Draco taeniopterus* | 77.2 | 27.1 | 135 | * | – | 1, 2 |
| ZFMK 21295 | *Draco timorensis* | 62.3 | 20.0 | 127 | * | – | 1, 2 |
| ZFMK 96689 | *Draco timorensis* | 76.1 | 22.2 | 122 | * | – | 1, 2 |
| ZFMK 20895 | *Draco volans* | 76.7 | 23.1 | 126 | * | – | 1, 2 |
| ZFMK 20896 | *Draco volans* | 66.7 | 20.0 | 131 | * | – | 1, 2 |
| ZFMK 20897 | *Draco volans* | 64.1 | 20.5 | 130 | * | – | 1, 2 |
| ZFMK 96314 | *Draco* sp. | 58.4 | 17.9 | 121 | * | – | 1, 2 |
| ZFMK 54834 | *Aphaniotis acutirostris* | 51.4 | 20.4 | n/a | – | – | 0 |
| ZFMK 51699 | *Aphaniotis fusca* | 64.8 | 28.0 | n/a | – | – | 0 |
| ZFMK 54833 | *Aphaniotis fusca* | 64.1 | 26.1 | n/a | – | – | 0 |
| ZFMK 20794 | *Bronchocela cristatella* | 94.0 | 32.8 | n/a | – | – | 0 |
| ZFMK 19277 | *Bronchocela jubata* | 129.6 | 52.8 | n/a | – | – | 0 |
| ZFMK 87062 | *Bronchocela vietnamensis* | 96.6 | 36.5 | n/a | – | – | 0 |
| ZFMK 96231 | *Calotes bachae* | 87.7 | 28.2 | n/a | – | – | 0 |
| ZFMK 96232 | *Calotes bachae* | 68.7 | 21.9 | n/a | – | – | 0 |
| ZFMK 96703 | *Calotes bachae* | 80.7 | 26.2 | n/a | – | – | 0 |
| ZFMK 31783 | *Calotes ceylonensis* | 78.9 | 22.3 | n/a | – | – | 0 |
| ZFMK 31784 | *Calotes ceylonensis* | 73.9 | 23.5 | n/a | – | – | 0 |
| ZFMK 92035 | *Calotes emma* | 110.5 | 38.2 | n/a | – | – | 0 |
| ZFMK 92028 | *Calotes mystaceus* | 82.6 | 28.0 | n/a | – | – | 0 |
| ZFMK-H 8497 | *Calotes rouxi* | 60.8 | 22.9 | n/a | – | – | 0 |
| ZFMK-H 13891 | *Calotes rouxi* | 59.1 | 19.9 | n/a | – | – | 0 |
| ZFMK-H 14258 | *Calotes rouxi* | 54.8 | 19.3 | n/a | – | – | 0 |
| ZFMK 16503 | *Calotes versicolor* | 86.6 | 29.5 | n/a | – | – | 0 |
| ZFMK 16504 | *Calotes versicolor* | 81.0 | 26.6 | n/a | – | – | 0 |
| ZFMK 849 | *Ceratophora stoddartii* | 75.1 | 25.3 | n/a | – | – | 0 |
| ZFMK 21480 | *Cophotis ceylanica* | 67.4 | 17.1 | n/a | – | – | 0 |
| ZFMK 46198 | *Dendragama boulengeri* | 75.0 | 24.9 | n/a | – | – | 0 |
| ZFMK 48896 | *Harpesaurus beccarii* | 78.8 | 20.7 | n/a | – | – | 0 |
| ZFMK 41567 | *Japalura polygonata* | 70.6 | 23.7 | n/a | – | – | 0 |
| ZFMK 20821 | *Japalura swinhonis* | 70.5 | 22.0 | n/a | – | – | 0 |
| ZFMK 95271 | *Mantheyus phuwuanensis* | 42.2 | 18.7 | n/a | – | – | 0 |
| ZFMK 74733 | *Oriotiaris* sp. | 36.7 | 12.1 | n/a | – | – | 1, 2 |
| ZFMK 85370 | *Phoxophrys borneensis* | 50.5 | 14.8 | n/a | – | – | 0 |
| ZFMK 20790 | *Pseudocophotis sumatranus* | 77.3 | 23.9 | n/a | – | – | 0 |
