# Supplementary figures and images for: How lizards fly: A novel type of wing in animals
Source: PLoS One. 2017 Dec 13;12(12):e0189573. doi: 10.1371/journal.pone.0189573 (PMC5728497; doi:10.1371/journal.pone.0189573)

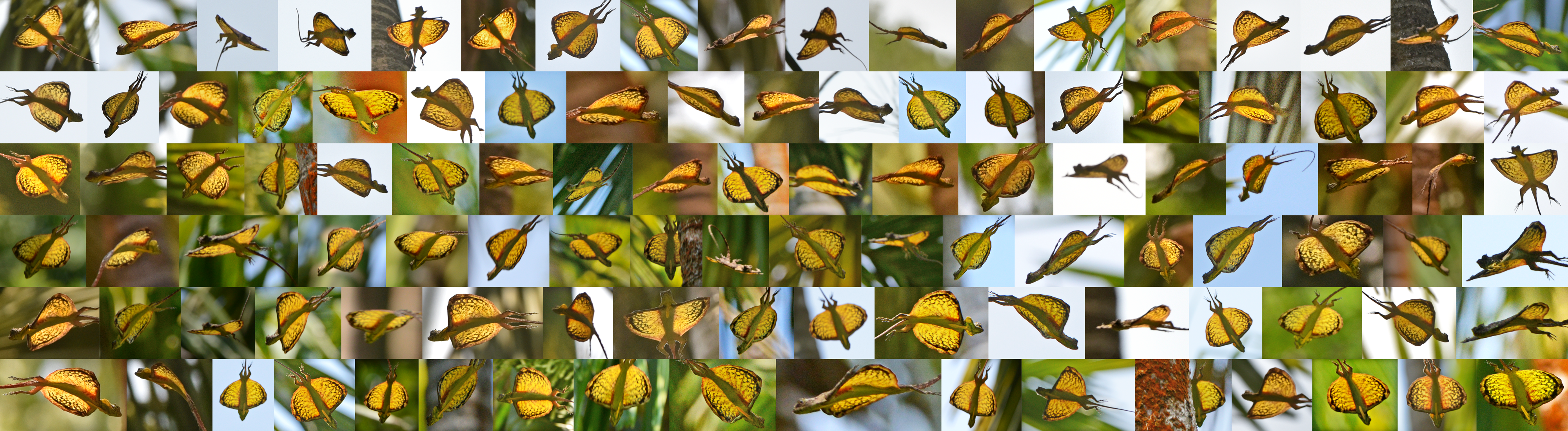

Supplement: S1 Fig — (TIF) [file pone.0189573.s002.tif]

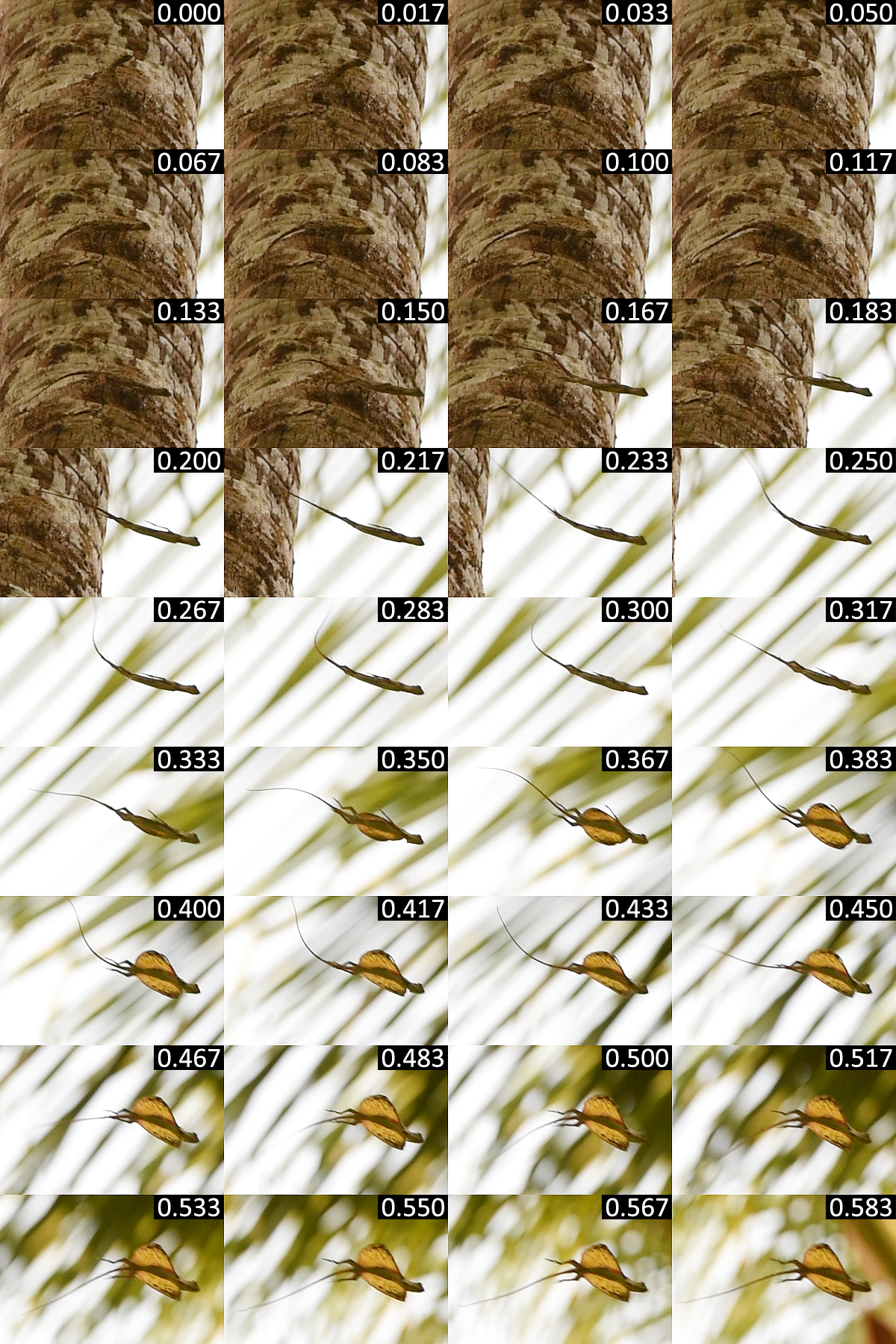

Supplement: S2 Fig — Consecutive still frames from a video (S2 Video) recorded at 60 frames/s. Numbers in the upper right corner are the running time given in seconds. (TIF) [file pone.0189573.s003.tif]

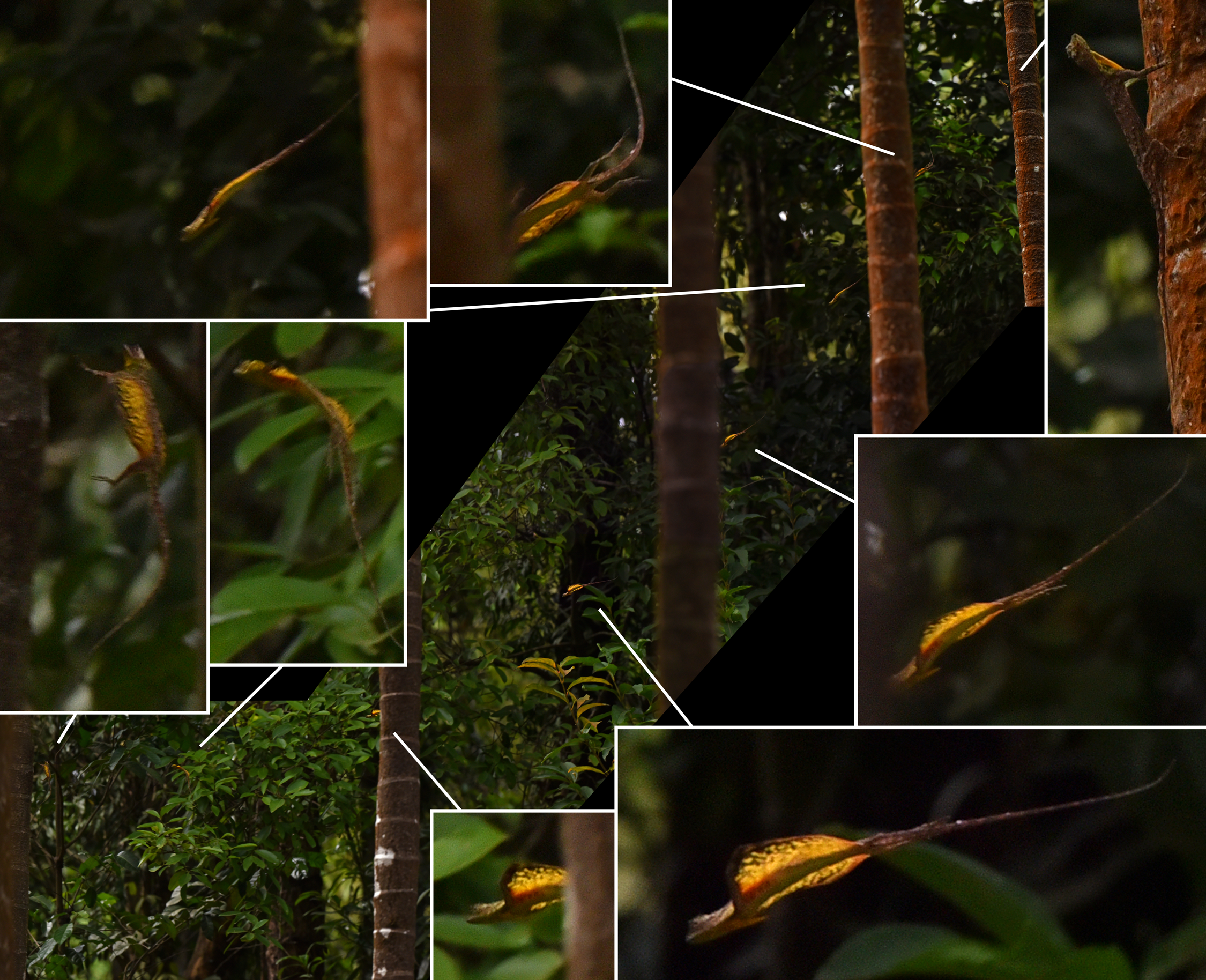

Supplement: S3 Fig — Composite image composed of photos taken at a rate of 6.5/s, showing the different glide phases, and details of the corresponding individual photos. The lizard jumps from a tree (upper right corner) and forms its composite wing. Subsequently, the trajectory becomes more horizontal. Before landing, the aerofoil is orientated upwards and the forward speed is reduced. The lizard approaches the landing point in a horizontal trajectory, hitting it with hands and feet almost simultaneously. (TIF) [file pone.0189573.s004.tif]

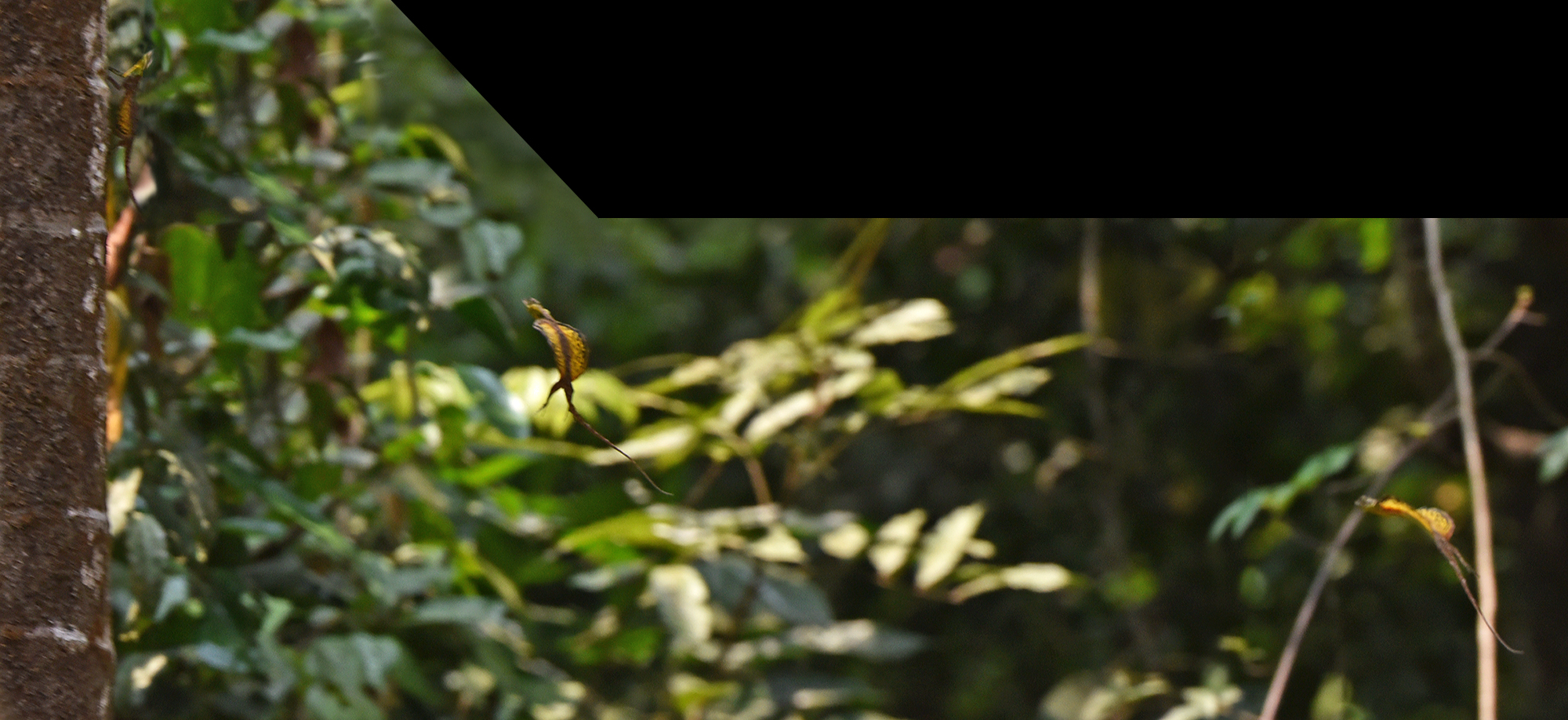

Supplement: S4 Fig — Hands with the lateral parts of the leading edge are raised above the horizontal body plane, causing a change of the angle of attack (right). The trajectory first becomes horizontally than turns even upwards. Eventually, the aerofoil is brought in a near vertical position and the forward speed is reduced. Note the forelimbs being connected to the leading edge of the patagium (right and middle). (TIF) [file pone.0189573.s005.tif]

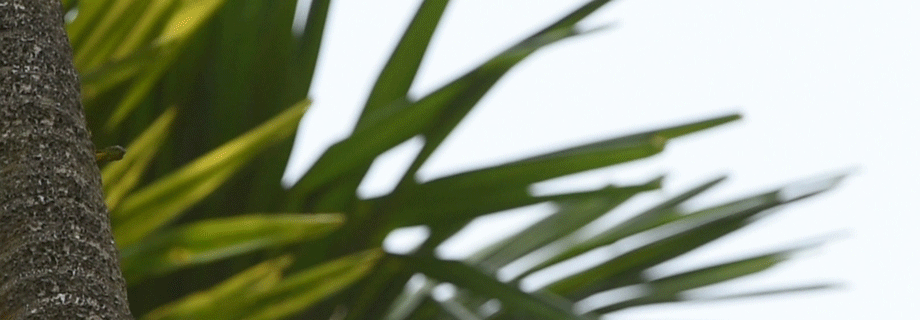

Supplement: S1 Animation — Animation in “Graphics Interchange Format” (GIF) using the still frames from S1 Video slowed down tenfold. See also Fig 1. (GIF) [file pone.0189573.s009.gif]

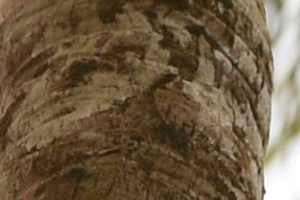

Supplement: S2 Animation — Animation in GIF using the still frames from S1 Fig (S2 Video) slowed down tenfold. (GIF) [file pone.0189573.s010.gif]

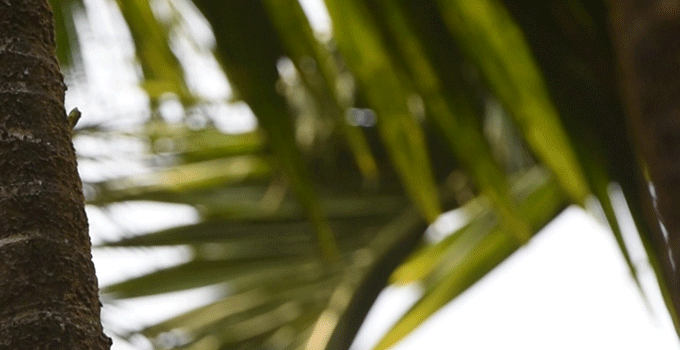

Supplement: S3 Animation — Animation in GIF using the still frames from S3 Video slowed down tenfold. (GIF) [file pone.0189573.s011.gif]

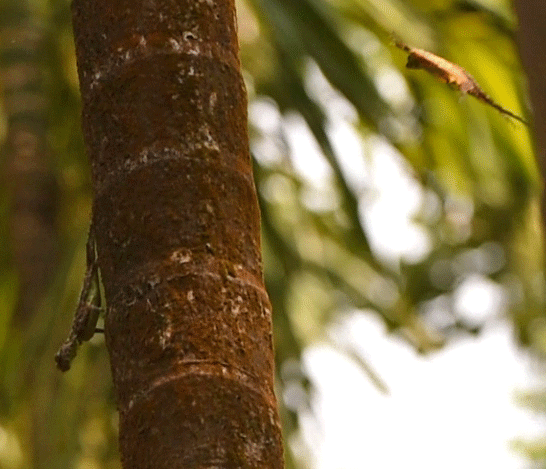

Supplement: S4 Animation — Animation in GIF using the still frames from a video recorded at 60 frames/s, slowed down twentyfold. See also Fig 4. (GIF) [file pone.0189573.s012.gif]
